# Efficiency of genomic selection in an established commercial layer breeding program

**DOI:** 10.1186/1297-9686-45-29

**Published:** 2013-07-31

**Authors:** Florian Sitzenstock, Florence Ytournel, Ahmad R Sharifi, David Cavero, Helge Täubert, Rudolf Preisinger, Henner Simianer

**Affiliations:** 1 Department of Animal Sciences, University of Göttingen, 37075 Göttingen, Germany; 2 Lohmann Tierzucht GmbH, 27472 Cuxhaven, Germany; 3 Vereinigte Informationssysteme Tierhaltung w.V. (vit), 27283 Verden, Germany

## Abstract

**Background:**

In breeding programs for layers, selection of hens and cocks is based on recording phenotypic data from hens in different housing systems. Genomic information can provide additional information for selection and/or allow for a strong reduction in the generation interval. In this study, a typical conventional layer breeding program using a four-line cross was modeled and the expected genetic progress was derived deterministically with the software ZPLAN+. This non-genomic reference scenario was compared to two genomic breeding programs to determine the best strategy for implementing genomic information in layer breeding programs.

**Results:**

In scenario I, genomic information was used in addition to all other information available in the conventional breeding program, so the generation interval was the same as in the reference scenario, i.e. 14.5 months. Here, we assumed that either only young cocks or young cocks and hens were genotyped as selection candidates. In scenario II, we assumed that breeders of both sexes were used at the biologically earliest possible age, so that at the time of selection only performance data of the parent generation and genomic information of the selection candidates were available. In this case, the generation interval was reduced to eight months. In both scenarios, the number of genotyped male selection candidates was varied between 800 and 4800 males and two sizes of the calibration set (500 or 2000 animals) were considered. All genomic scenarios increased the expected genetic gain and the economic profit of the breeding program. In scenario II, the increase was much more pronounced and even in the most conservative implementation led to a 60% improvement in genetic gain and economic profit. This increase was in all cases associated with higher breeding costs.

**Conclusions:**

While genomic selection is shown to have the potential to improve genetic gain in layer breeding programs, its implementation remains a business decision of the breeding company; the possible extra profit for the breeding company depends on whether the customers of breeding stock are willing to pay more for improved genetic quality.

## Background

Breeding of layers for commercial egg production is an international business and is dominated by a few companies. The marketable product of these companies is the breeding stock that subsequently is used to produce laying hens in various production systems. In egg production, the profit margins are extremely tight, especially with the increasing feed prices and changing production systems [1]. The strong competition on the market for laying hens also contributes to a substantial economic pressure. In such a highly competitive market, the strategy of a breeding company with regard to allocation of resources needs to be continuously optimized and monitored in order to maintain its competitiveness and market share on the global market.

Breeding of commercial laying hens is based on the pool of nucleus lines of a breeding company. Selection is done within closed purebred lines and is based on a comprehensive phenotyping scheme, both in pure line birds under highly standardized housing conditions, and in crossbred hens under housing conditions that are closer to the production environment of the end product. The age at selection in both sexes depends on the age when the performance of the hens is recorded. In current breeding schemes, cocks and hens are usually selected at one year of age or slightly older. Biologically, both cocks and hens could be used for reproduction much earlier since they achieve their sexual maturity at about five months of age.

The benefit of genomic selection over conventional selection strategies was first reported for dairy cattle. This expected benefit is due to the reduction in the generation interval, the increase in accuracies of the estimated breeding values of young bulls and bull dams, and the reduction in costs for progeny testing young bulls [2,3]. Based on these theoretical findings, genomic selection was rapidly implemented in dairy cattle breeding programs [4].

The breeding structures and the biological conditions in layer breeding programs differ strongly from those in dairy cattle breeding programs in many aspects. Therefore, optimum breeding strategies for the implementation of genomic selection in layer breeding programs must be designed and their comparative advantages must be assessed. Some studies showed the possibility to reduce generation interval in layer breeding programs by implementing genomic selection e.g. [5,6].

Among farm animals, the genome of the chicken was the first to be completely sequenced [7]. By 2006, Abasht et al. [8] had already reviewed 50 articles on quantitative trait loci identified in chicken. However, in practical breeding programs, marker-assisted selection has been implemented only for a few traits, e.g. fishy taint in brown layers [9] and susceptibility to Marek´s disease [10]. Currently, an array comprising about 60 000 single nucleotide polymorphisms (SNP) (60k Illumina SNP BeadChip) is available for the chicken [11].

The objectives of this study were to present a methodology for the economic evaluation and optimization of genomic selection programs. Towards this aim, we evaluated the potential benefits of genomic selection approaches in an existing layer crossbreeding program as an example for breeding programs with complex structures and breeding goals. A conventional breeding program based on a four-line cross was used as a reference. We implemented two genomic selection scenarios; in scenario I, genomic information was used in addition to the phenotypic information available at selection; in scenario II, early selection based on a combination of parental and genomic information was used. In both scenarios, additional parameters such as the size of the calibration set and the number of genotyped selection candidates were varied. We discuss the results both with respect to expected genetic progress on the level of single or combined traits, as well as on an economic scale. Here, the balance of expected costs and returns is delicate and different aspects of the practical implementation of genomic selection in commercial layer breeding programs will be addressed.

## Methods

### Modeling software

The software ZPLAN+ [12] was used to compare conventional crossbreeding programs with breeding programs using genomic information. This software allows modeling of all relevant breeding structures, while taking all relevant biological, technological and economic parameters for complex breeding programs into account. ZPLAN+ is based on the gene flow theory [13], the selection index by Hazel and Lush [14], as well as on a complex modeling of costs and returns. ZPLAN+ is based on a similar conceptual approach as the software ZPLAN [15] but has many additional features (such as the possibility to model genomic information) and a more appropriate web-based user interface. Like ZPLAN, it deterministically calculates the expected genetic trend and the discounted economic gain and profit over a defined planning horizon. The monetary results in ZPLAN+ are standardized to an animal unit and are given per year. We defined the selected animals in the four lines and all grandparental animals as basis for the standardization, which resulted in a total number of animals equal to 127 640.

### Input parameters

To model a breeding program in ZPLAN+, input parameters to define the biological aspects and the breeding processes must be specified. All traits that are either recorded and/or part of the breeding goal must be specified. For each trait, heritability, phenotypic standard deviation, economic value and genotypic correlations to all other traits must be given. Phenotypic correlations are only required between the traits that are actually measured for the birds in the same environment. The breeding goal is implicitly defined by assigning economic weights to all or a subset of the defined traits.

For each trait, the different information source groups (e.g. full sib groups) and the traits that are recorded for these must be defined. This includes specifying the number of individuals in the group, the number of repeated measurements of each trait, and the additive-genetic relationships within the group and with the selection candidates.

The animals in the breeding program are divided into selection groups. A selection group is a group of animals having the same sex and ancestry. For each selection group, the following information has to be provided: the breeding goal for the group, the information source groups available for each selection candidate, the number of tested and selected animals, the variable costs for each tested animal, the age at first reproduction, and the productive lifetime.

ZPLAN+ provides a comprehensive set of results. For each selection path, the genetic gain is computed as the product of the accuracy of the selection index, the selection intensity and the standard deviation of the breeding goal in this path of selection. The discounted variable costs are calculated across the selection groups. Discounted variable costs plus discounted fixed costs result in total costs. The discounted return of a selection path is obtained as the monetary genetic gain weighted by its corresponding Standardised Discounted Expression (SDE) value. The SDE value of a selection path includes the proportion of animals that realize the genetic gain, the point in time of realization, and the amount of the genetic gain provided by this selection group over all generations within the planning horizon. The total discounted return is the sum of returns over all selection paths. The profit is the total discounted return minus the total discounted costs and is expressed per animal unit in the breeding program.

The general concept of ZPLAN+ is based on assessing the accumulated effect of a single round of selection; therefore a reduction in the genetic variance with recurrent selection, the so-called Bulmer effect [16], is not modeled explicitly. However, since the genetic parameters are taken from an ongoing breeding program, the reduction in genetic variance due to the Bulmer effect is implicitly accounted for.

### Conventional breeding program

**Figure 1:**
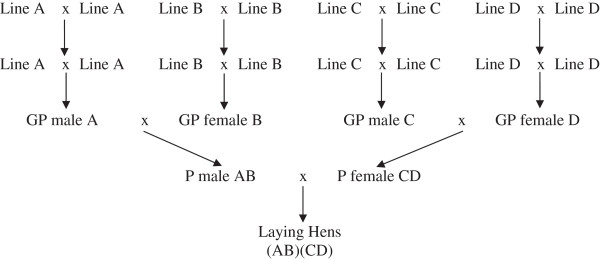
**Schematic structure of the crossbreeding program.** GP = Grand Parents; P = Parents.

A commercial layer crossbreeding program with four nucleus lines (A, B, C and D) was modeled in ZPLAN+ for a planning period of ten years (Reference Scenario). In these four lines, the selection process relied on a combination of information measured in the purebred birds at the breeding unit and on information captured from crossbred half-sibs under commercial conditions. From the pure lines, the grandparent generation (grandparents of the production hens) was hatched in a first multiplication step. In the parent generation, the cocks were a cross of lines A and B, while the hens were a cross of lines C and D. This resulted in 500 000 cocks and 5.10^6^ hens in the parental generation. The cross of this parental generation finally produced 500.10^6^ laying hens for egg production. The complete breeding scheme is presented in Figure 1.

Each pure line comprised the same number of animals. In each line and generation, 600 hens and 60 cocks were used. A pre-selection after rearing was carried out on the basis of parental information. Four thousand female chicks to be housed on the breeding farms were selected from 4800 reared chicks. The 4000 selected hens were then tested in single cages over 32 weeks from age 20 to 51 weeks, to finally select 500 hens based on an index combining parental information with own, full- and purebred half-sib performance. In addition, 1500 hens per line were tested in group cages (three full- or half-sib hens per cage), from which an additional 100 hens per line were selected.

The selection of 60 cocks per line from 800 male chicks was based on parental information plus the following information on relatives:

•8 purebred full-sibs and 65 pure-bred half-sibs in single cages,

•4 purebred full-sibs and 20 pure-bred half-sibs in group cages,

•30 crossbred half-sibs in a practical environment.

To produce the 30 crossbred half-sibs, cocks of lines A and B were mated with hens of lines C or D, resulting in crossbred hens of genotype AC, AD, BC or BD, which were tested in the practical environment.

The 60 selected cocks and 600 selected hens per line were used to produce the new purebred generation. All hens, including those not selected, were maintained during the entire production cycle to have information on the late laying performance for the next generation.

The breeding goal comprised performance and functional traits. The selection indexes for the purebred hens in single or group cages and the hens in the practical environment included 22 traits (Table 1). The laying performance was split in four time periods. Table S1 [See Additional file 1: Table S1] provides the relative economic weights (ew), phenotypic standard deviations, heritabilities, and genetic and phenotypic correlations of all 22 traits. These parameters were based on a breeding program for laying hens from the Lohmann Tierzucht GmbH. Note that the sign of the economic weight indicates the desired direction of genetic change in the respective trait (e.g. for feed consumption and mortality, a negative economic weight indicates that a numerical reduction of the trait level was desirable). While ZPlan+ requires economic weights in € per genetic standard deviation, the values in Table S1 [See Additional file 1: Table S1] are scaled to 100 to allow a direct assessment of the relative values of traits in the breeding goal.

**Table 1:** Traits recorded in the different housing systems

	**Trait unit**	**Single cage**	**Group cage**	**Practical environment**
Laying performance 1	%	X	X	X
Laying performance 2	%	X	X	X
Laying performance 3	%	X	X	X
Laying performance 4	%	X		X
Egg weight	g	X	X	X
Feed consumption	g	X		
Egg shell strength	N	X	X	X
Hatchability	%	X*		
Mortality	1 or 0	X	X	X
Feathering quality	Scale 1 – 9		X	

*only in C and D.

The variable costs of rearing a cock or a hen over a period of 20 weeks were assumed to be €11 per animal. During production, daily feeding costs resulted from a feed consumption of 0.11 kg per day at a price of €0.3 per kg. For each cock and hen, additional costs for the cage unit (€5) and animal care (€5) were assumed. Performance testing caused additional costs of €5 per hen. Since it was difficult to quantify additional fixed costs that may be associated with decisions on the breeding structures (e.g. the cost of a new performance test unit when the population size is increased), we included no fixed costs in the reference scenario. This has no influence on the comparison of alternative scenarios if we assume that they are indifferent with respect to the population size. However, it must be kept in mind that fixed costs have to be paid from the resulting profit. The considered time frame was set to ten years (i.e. ten breeding cycles). The interest rate was set to 7% for discounted costs and 2% for discounted returns.

In order to evaluate and compare the different scenarios for the breeding programs, results reported include genetic gain in each trait, total monetary genetic gain, generation interval, and the discounted economic parameters of returns, costs and profit. To allow for a better comparison, we set the values obtained for the reference scenario (conventional breeding program) to 100% and expressed the results obtained with the alternative genomic scenarios relative to these reference values.

### Genomic breeding programs

In ZPLAN+, a genomic counterpart trait for each conventional trait is defined to be the genomic estimated genomic breeding value (GEBV) for that trait, based on the given calibration set size. The background for implementing genomic information in the selection index on this basis was developed by Dekkers [17] and modified by Haberland et al. [18]. This approach requires the correlation of the true and the GEBV to define for every genomic trait $r_{Q\hat{Q}}$. This is done using the approach by Erbe et al. [19] based on an equation derived by Daetwyler et al. [20,21]:

$$r_{Q\hat{Q}}=w\sqrt{\frac{Nr_{\mathrm{TI}}^{2}}{Nr_{\mathrm{TI}}^{2}+M_{e}}},$$

where *N* is the size of the calibration set, *w* is a calibration factor, $r_{\mathrm{TI}}^{2}$ is the reliability of the GEBV of the animals used in the calibration set, and *M*_*e*_ is the number of independently segregating chromosome segments, which was derived by Goddard [22] as:

$$M_{e}=\frac{2N_{e}L}{\ln\left(4N_{e}L\right)},$$

where *N*_*e*_ is the effective population size, which was assumed to be 60 per line and *L* is the length of the genome in Morgan which was set to 32, based on Groenen et al. [23], leading to *M*_*e*_ = 429.

The calibration factor *w* reflects the accuracy of GEBV that is hypothetically obtained with a calibration set of infinite size using the given SNP density. Erbe et al. [19] empirically determined this factor to be *w* ~ 0.9 for different traits in dairy cattle and for GEBV from a 50k SNP chip. Since for layers, this quantity is unknown, we also assumed *w* ~ 0.9, but it will be necessary to determine *w* from empirical data once they are available.

We modeled two different genomic scenarios:

Scenario I: in this scenario, the genomic information of cocks or both sexes was added to all other information that is available in the reference scenario but all selection decisions were made at the same time as in the reference scenario.

Scenario II: this scenario assumed that selection takes place at the biologically earliest possible stage, so that only parental and genomic information is available for different numbers of cocks and for all 6300 reared hens. Selected animals were used for breeding by eight months of age. For re-calibration of the genomic information and the production of the grandparental generation, hens were kept and fully performance tested until 72 weeks of age.

In both genomic scenarios, the number of genotyped cocks was varied between 800 and 4800 animals per purebred line in steps of 800, while the number of selected cocks was kept constant at 60. While in the conventional scheme all full-sibs have the same EBV and drawing one animal from a group at random is the best one can do, it is possible to select between full-sibs when GEBV are available. As an alternative for scenario I, we assumed that all 6300 reared hens were also genotyped and this additional information was used for dam selection.

The variable costs for genotyping an animal were assumed to be €150. The total cost to implement GEBV estimation was €150 multiplied by the number of animals in the calibration set, which was assumed to equal 500 or 2000 animals. These costs were fixed costs for the genomic breeding programs and were divided by the time horizon of ten years. All incurred variable costs were accounted for in the same way as in the reference scenario.

## Results

### Reference scenario

The generation interval in the reference scenario was 14.5 months for each line. The accuracy of the selection index at the time of selection was *r*_*TI*_ = 0.51 for cocks, *r*_*TI*_ = 0.53 for hens tested in single cages, and *r*_*TI*_ = 0.51 for hens tested in group cages. The genetic gain per year for each trait is in Table 2.

**Table 2:** Genetic gain per year in the reference scenario, expressed in genetic standard deviations

**Housing system**	**Trait**	**Genetic gain**
**Single cage**	Laying performance 1	0.074
	Laying performance 2	0.389
	Laying performance 3	0.497
	Laying performance 4	0.422
	Egg weight	0.116
	Feed consumption*	0.033
	Egg shell strength	0.240
	Hatchability	0.025
	Mortality*	−0.116
**Group cage**	Laying performance 1	−0.008
	Laying performance 2	0.215
	Laying performance 3	0.199
	Egg weight	0.116
	Egg shell strength	0.166
	Mortality*	−0.041
	Feathering quality	0.116
**Practical environment**	Laying performance 1	0.091
	Laying performance 2	0.174
	Laying performance3+4	0.166
	Egg weight	0.116
	Egg shell strength	0.248
	Mortality*	−0.149

*negative genetic gains are in the desired direction.

The discounted return per animal unit in the reference scenario was €282.17 per year. The variable costs were €17.19 per animal unit, which resulted in a profit of €264.98 per animal unit. With 5.10^6^ laying hens on the reproduction level realizing the genetic progress obtained in the breeding nucleus, we have 3917 production hens per animal unit in the breeding nucleus, resulting in a profit of €0.07 per laying hen. Note that this profit does not include fixed costs, so these will have to be covered from this profit.

### Scenario I

**Figure 2:**
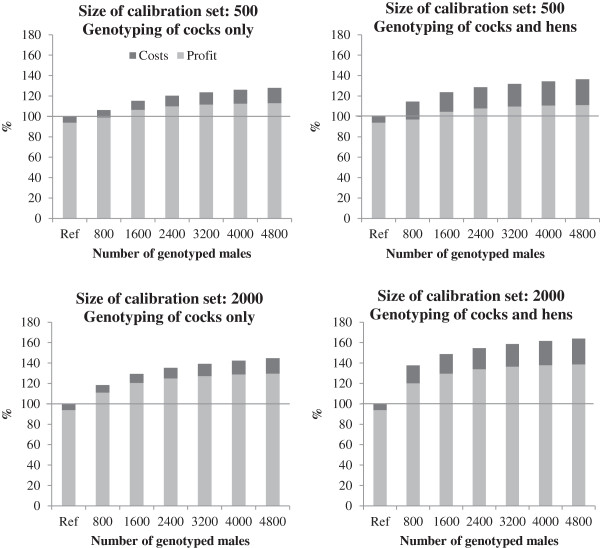
**Profit and costs (sum = returns) for Scenario I.** Results with genotyping of cocks (left panels) and both cocks and hens (right panels) are compared to the reference scenario (Ref; returns set to 100%) for two calibration set sizes.

In this scenario, the genomic information was added in the reference scenario at the time of selection. Neither the generation interval nor the costs for the performance testing of the hens were reduced. The returns increased with the number of tested cocks and with an increasing number of animals in the calibration set (Figure 2). Total costs scaled with the number of genotyped animals. The number of animals in the initial calibration set had no significant impact on the total costs, since these costs are distributed over the whole period considered, i.e. ten years and over all animals that receive the accumulated genetic gain. Compared to the reference scenario, the profit was higher for all numbers of tested cocks (€278.93 – €391.37 per animal unit) although there was a decreasing marginal increase in profit with increasing numbers of genotyped cocks. The latter is because adding more cocks increases genotyping costs linearly but the extra benefit from increasing selection intensity increases at a lower rate. With a calibration set of 500 animals, genotyping both sexes resulted in a marginally lower profit than genotyping cocks only.

**Figure 3:**
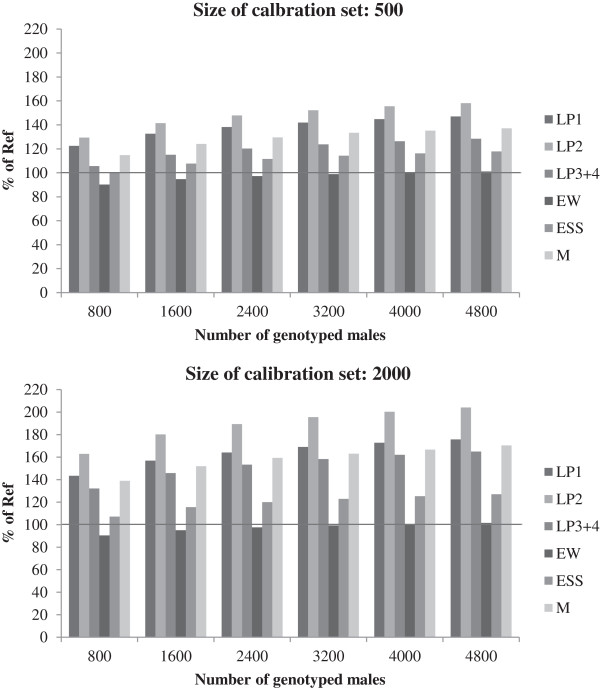
**Genetic gain for individual traits in crossbred hens in the practical environment for Scenario I.** LP1: laying performance 1, LP2: laying performance 2, LP3+4: laying performance 3 and 4, EW: egg weight, ESS: egg shell strength and M: mortality; genetic gain relative to the reference scenario (set to 100%) with different numbers of genotyped cocks and different sizes of the calibration set.

To compare the expected genetic gain for individual traits between scenarios, we used the six traits of the crossbred hens in the practical environment, since this is the type of production that is closest to the production system for which the hens are selected. In Figure 3, the predicted genetic change for these six traits is shown relative to the predicted change for the reference scenario. The gain for laying performance increased for all laying periods, with the highest gain for the second period, for which the genetic gain was doubled when a calibration set of 2000 cocks was used and 4000 cocks were genotyped for selection. Genetic gain in egg weight decreased slightly when only a few cocks were genotyped for selection and reached the same level as in the reference scenario only when 4000 or more cocks were genotyped for selection. With 2000 animals in the calibration set, the genetic gain was greater than with 500 animals but ranking of traits remained the same. In all cases, a considerable genetic improvement in mortality was observed. Adding the genotyping of hens increased genetic gain for all traits except egg weight (data not shown). In particular, genetic gains in laying performance and mortality benefitted from the additional genomic information. The additional increase in egg shell stability was only marginal and the genetic gain in egg weight was lower than when only the cocks were genotyped.

### Scenario II

**Figure 4:**
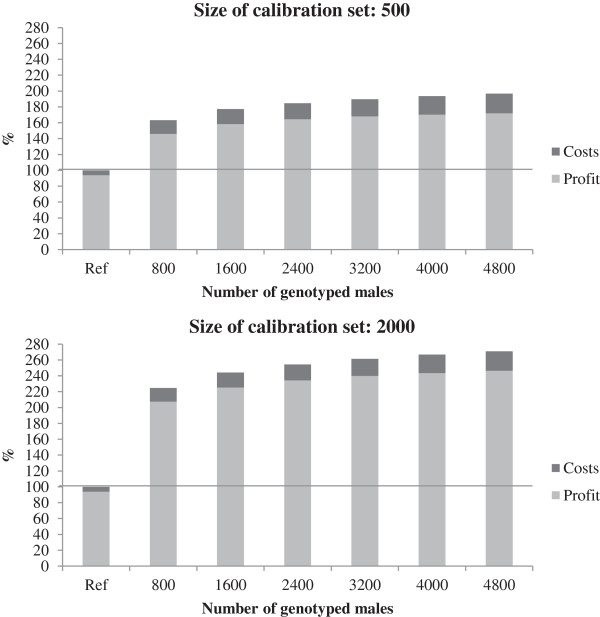
**Profit and costs (collectively: return) in Scenario II.** Genomic information of both sexes in relation to the return of the reference scenario (Ref) with different numbers of tested cocks and different sizes of calibration set.

In scenario II, the generation interval was reduced to eight months. By assuming that performance testing of hens continued after selection, the costs related to performance testing were not reduced. The returns, costs and profit are shown relative to the reference scenario in Figure 4. The figure shows that with a calibration set of 2000 animals, the returns can be doubled, even with very few (800) male animals genotyped for selection. With the initially more realistic number of 500 animals in the calibration set, the returns increased by 60 to over 100% with 800 to 4800 cocks genotyped. The costs increased with increasing numbers of animals genotyped. With the lowest number of genotyped cocks, the costs were three times greater than in the reference scenario (€48.84 per animal unit) and with the highest number of genotyped cocks, they were more than quadrupled (€69.80 per animal unit).

**Figure 5:**
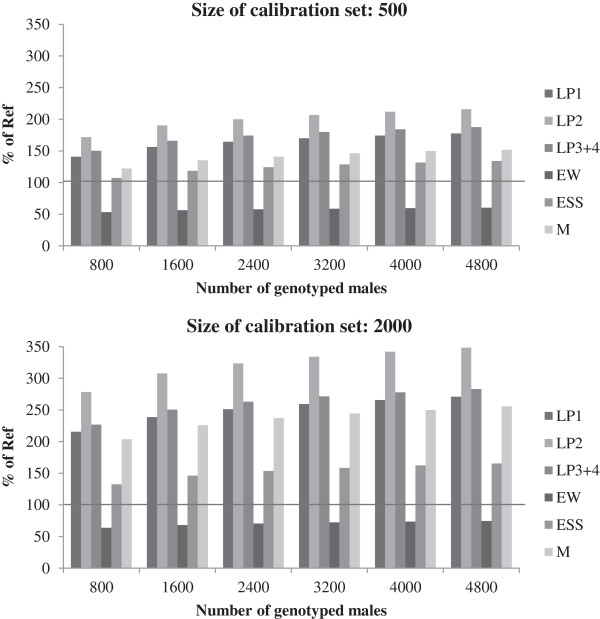
**Genetic gain in crossbred hens in practical environment in Scenario II for different traits.** LP1: laying performance 1, LP2: laying performance 2, LP3+4: laying performance 3 + 4, EW: egg weight, ESS: egg shell strength and M: mortality; genomic information of the cocks in relation to the reference scenario with different numbers of tested cocks and different sizes of calibration set.

The genetic gain in the six traits of the crossbred hens in the practical environment is shown in Figure 5. While the genetic progress was substantially increased (partly doubled or tripled) for most traits, the genetic gain in egg weight was lower than in the reference scenario. In many breeding programs, however, egg weight is considered a trait under stabilizing selection (i.e. neither an increase nor a decrease of the trait level is desired), so some reduction in genetic gain may be acceptable for this trait. This result for egg weight may also be a consequence of aiming at a higher laying performance with lower feed consumption, which has consequences on egg size.

## Discussion and conclusions

In general, this report demonstrates that the complete breeding structures of a conventional commercial crossbreeding program for laying hens can be comprehensively modelled with ZPLAN+. Although we simplified the breeding structures to some extent compared to what is implemented in a real breeding program, we still modelled 23 selection classes and 18 selection index variants in the different scenarios. ZPLAN+ allows for such a complexity by having, e.g., no limit on the number of traits or information groups in the selection index module. However, the more complex a scenario becomes, the more challenging it is to define admissible parameters, e.g. positive definite variance-covariance matrices. In this case study, we showed that ZPLAN+ can handle breeding programs of different dimensions and complexity.

Based on a conventional reference scenario, we evaluated different approaches to implement genomic information in the breeding strategies for laying hens. In scenario I, the genomic information was used as additional information. In scenario II, the selection in the purebred lines was based only on pedigree and genomic information, which allows the generation interval to be shortened substantially.

Genomic information was shown to have a positive effect on monetary genetic gain in all scenarios. The size of the calibration set and the accuracy of the genomic information were found to have a large impact on the benefits from genomic selection. The theoretical background for this dependency was given by Dekkers [17], Daetwyler et al. [20] and Erbe et al. [19], and practical calculations for other farm animals with ZPLAN or ZPLAN+ show the same trend [3,24,25]. Over time, the number of animals available for recalibration will increase, which is expected to lead to a higher accuracy of genomic predictions, although the number of additional cocks with performance tested progeny will be small (60 per year and line). This study focused on the implementation and short-term effects of incorporating genomic information in a layer breeding program in the process transition from traditional to genomic breeding programs. Therefore, we did not model the dynamics of changing population sizes (in particular, training set sizes) over time. In scenario II, the advantage of genomic selection was particularly evident, because genomic information was the main basis for selection, such that the generation interval could be reduced substantially.

In the reference scenario, selection of cocks and hens was carried out at one year of age, after the hens were performance tested. This led to a generation interval of 14.5 months. Since in scenario I, genomic information was only added as additional information, the generation interval was the same as in the reference scenario. In scenario II, the selection of cocks and hens relied only on genomic information and on the performance of the mother and of the full-sibs and half-sibs of the father. This made it possible to reduce the generation interval to the biological limit of about eight months, because the time of selection did not depend on performance information of contemporary hens. A reduction in generation interval is linked to an increase in the coefficient of inbreeding per year. However, several studies have reported that increase in inbreeding has only a small negative effect on the genetic gain of breeding goal traits in poultry e.g. [26,27]. The effects of reducing generation interval were found in dairy cattle too, where the use of genomic information replaces progeny information, so that young bulls can be used immediately [2,3], which can reduce the variable costs per proven bull substantially. In conventional dairy cattle breeding programs, there are substantial costs related to housing and feeding the bulls for several years until the performance tests of their daughters are available. In addition, because a substantial proportion of the cow population is mated to test bulls with an inferior average breeding value compared to progeny tested bulls, this causes economic losses, which, together with the housing costs, add up to about €25 000 per tested bull. In layer breeding programs, the costs of keeping a cock are marginal and the performance information of the hens is necessary anyway for a recalibration of the GEBV. In addition, hens are used to produce the grandparental generation as well as the crossbred hens for performance testing. Thus, the benefit of scenario II in layers is mainly due to the reduced generation interval rather than to a significant reduction of breeding program costs, as may be the case for dairy cattle [2]. In pigs, Henryon et al. [28] showed that only a small proportion of the selection candidates preselected based on phenotype information need to be genotyped to realize most of the benefits. This approach cannot be used to reduce the generation interval in layers because in layers phenotype is measured after breeding age. However in scenario I, such an approach could reduce genotyping costs.

The increase in genetic gain in the genomic scenarios was associated with a strong increase in costs, mainly for genotyping. The greater expected returns could offset these increased costs, but it has to be noted that the costs are real, while the benefits are theoretical predictions and will also only turn into a realized economic benefit if improved breeding products can be sold at a higher market price, or if the genetic superiority increases market share in a competitive market.

The genotyping costs for the initial calibration study only had a marginal effect in the calculations because they were distributed across 127 640 animal units over a time frame of ten years. However, the costs of genotyping the calibration set (for 2000 animals the genotyping costs are 300 000 €) results in a considerable investment to be made in order to obtain an expected competitive advantage over a considerable period (ten years), which may or may not materialize and generate an economic profit. If competing breeding companies also invest into a calibration study and establish a similar genomic selection scheme, then there will be no competitive advantage despite the fact that the investment was done. However, there would be a competitive disadvantage for the company if the investment had not been made.

A reduction in genotyping costs of the genomic selection scheme could be achieved by the use of low-density chips containing a subset (e.g. 3 k to 6 k) of SNPs from the 60 k chip. This would reduce the costs of genotyping of selection candidates in each generation and nearly all the genomic information content could be retrieved by imputation strategies [29,30] and only selected animals would need to be re-genotyped with the 60k chip. In dairy cattle, applying such an approach did not affect the quality of genomic information significantly [30].

The expected genetic gain in individual traits from genomic selection differed substantially between traits. In particular, in the case of egg weight, the genetic gain was similar or even reduced compared to the reference scenario, while for the other five traits, genetic gains increased with the amount of genomic information (size of the calibration set and number of genotyped selection candidates). The reasons for this were the low economic weight of egg weight in all housing systems and the negative genetic correlations of egg weight with many other traits, especially laying performance. This was particularly evident in scenario II, in which performance testing information from the present generation was not used for selection. The relative benefits from genomic selection were high for traits with a low heritability such as mortality. Functional traits are especially expected to benefit from the use of genomic information [31] but in our study, performance traits with a moderate heritability and higher economic weights in the breeding goal also benefitted from using genomic information. This was confirmed by Wolc et al. [32], who showed using real data that accuracy of the EBV increased when layers were selected on the basis of genomic information and no phenotypic information was available.

Our study provides a basis to optimize layer breeding programs from an economic point of view. In particular, not having to wait for performance information of the hens could boost the genetic trend and increase the expected monetary genetic gain. In addition, a combination of genomic information and available performance information from the present generation, as in scenario I, could increase the monetary genetic gain compared to the conventional breeding program.

For breeding companies, it is basically a business decision whether an investment in a genomic breeding program is expected to pay off through additional returns from a higher genetic quality of the marketable products, or an increase in the market share in a competitive market. On the one hand, market research has to assess the willingness of the direct customers to pay more for breeding stock of higher quality. Hen producers will make their final decision on the basis of whether egg producers are willing to pay a higher price for better laying hens. On the other hand, failing to adopt a major innovation in an oligopolic situation may lead to a major competitive disadvantage if the competitors implement the innovation and realize the benefits, combined with an appropriate communication strategy.

In conclusion, genomic selection can substantially increase the efficiency of breeding programs for layers, as previously reported for other livestock species. Breeding structures and biological conditions in layers do not allow for a cost reduction but genomic information can be used either to increase the accuracy at selection (scenario I) or to shorten the generation interval (scenario II). The latter was shown to be the much more profitable strategy. However, the decision of whether or not to implement a major technological innovation such as genomic selection in a competitive market with an oligopolic structure does not only depend on expected genetic gains and profits but is primarily a business decision, taking into account the perceived consumers’ willingness to pay for improved genetics, as well as the assumed technological innovation strategy of the main competitors.

## Competing interests

The authors declare that they have no competing interests.

## Authors’ contributions

FS was involved in the design of the study, carried out the modelling and calculations and drafted the manuscript. FY contributed to the design of breeding programs and calculations. AS, DC and RP participated in the design of the study and monitored the practical relevance of the results. HT developed and maintains the software. HS designed and monitored the study and worked on the manuscript. All authors read and approved the final manuscript.

## Supplementary Material

Additional file 1: Table S1 The data provided represent the relative economic weights, phenotypic standard deviations, heritabilities, and genetic and phenotypic correlations of all traits in the breeding goal. Click here for file
