# Supplementary material for: Efficiency of genomic selection in an established commercial layer breeding program
Source: Genet Sel Evol. 2013 Jul 31;45(1):29. doi: 10.1186/1297-9686-45-29 (PMC3750290; doi:10.1186/1297-9686-45-29)
Supplement: Additional file 1: Table S1 — The data provided represent the relative economic weights, phenotypic standard deviations, heritabilities, and genetic and phenotypic correlations of all traits in the breeding goal. [file 1297-9686-45-29-S1.docx]

**Table S1 Required parameters for all traits**

|  |  |  | **ew** | **SD** | **1** | **2** | **3** | **4** | **5** | **6** | **7** | **8** | **9** | **10** | **11** | **12** | **13** | **14** | **15** | **16** | **17** | **18** | **19** | **20** | **21** | **22** |
| --- | --- | --- | --- | --- | --- | --- | --- | --- | --- | --- | --- | --- | --- | --- | --- | --- | --- | --- | --- | --- | --- | --- | --- | --- | --- | --- |
| **Single cage** | **Laying performance 1** | **1** | 3 | 22.7 | **0.35** | 0.16 | -0.05 | -0.10 | -0.20 | -0.14 | -0.06 | 0.05 | 0.05 | 0.73 | 0.05 | 0.02 | -0.20 | -0.05 | -0.03 | -0.17 | 0.40 | -0.05 | -0.10 | -0.15 | 0.18 | 0.03 |
|  | **Laying performance 2** | **2** | 6 | 4.8 | 0.06 | **0.10** | 0.73 | 0.58 | -0.30 | 0.05 | -0.05 | 0.15 | -0.05 | 0.05 | 0.30 | 0.20 | -0.15 | -0.08 | -0.02 | 0.05 | 0.07 | 0.20 | 0.10 | -0.12 | -0.09 | -0.05 |
|  | **Laying performance 3** | **3** | 6 | 7.0 | -0.01 | 0.47 | **0.12** | 0.85 | -0.20 | 0.09 | -0.08 | 0.15 | -0.05 | -0.05 | 0.22 | 0.25 | -0.10 | -0.09 | -0.02 | 0.02 | 0.01 | 0.16 | 0.20 | -0.10 | -0.10 | -0.05 |
|  | **Laying performance 4** | **4** | 9 | 7.3 | -0.07 | 0.27 | 0.32 | **0.20** | -0.20 | 0.10 | -0.12 | 0.20 | -0.08 | -0.10 | 0.12 | 0.15 | -0.09 | -0.08 | -0.02 | 0.03 | -0.05 | 0.20 | 0.33 | -0.10 | -0.08 | -0.10 |
|  | **Egg weight** | **5** | 18 | 3.8 | -0.18 | -0.08 | -0.04 | -0.06 | **0.75** | 0.64 | -0.20 | -0.40 | 0.02 | -0.18 | -0.15 | -0.14 | 0.80 | -0.22 | 0.01 | -0.03 | -0.10 | -0.20 | -0.15 | 0.70 | -0.15 | -0.04 |
|  | **Feed consumption** | **6** | -12 | 10.0 | -0.05 | 0.08 | 0.07 | 0.01 | 0.43 | **0.50** | -0.05 | -0.24 | 0.01 | -0.05 | -0.08 | -0.05 | 0.55 | -0.01 | 0.00 | 0.00 | -0.10 | -0.10 | -0.05 | 0.45 | -0.05 | 0.00 |
|  | **Egg shell strength** | **7** | 7 | 7.0 | 0.05 | 0.13 | 0.11 | 0.10 | -0.12 | -0.04 | **0.35** | 0.08 | 0.02 | -0.10 | -0.09 | -0.08 | -0.22 | 0.80 | 0.03 | 0.08 | -0.03 | 0.08 | 0.12 | -0.21 | 0.75 | -0.04 |
|  | **Hatchability** | **8** | 2 | 25.8 | 0.05 | 0.13 | 0.09 | 0.07 | -0.25 | -0.13 | 0.09 | **0.26** | -0.01 | 0.06 | 0.05 | 0.05 | -0.52 | -0.07 | 0.00 | 0.00 | 0.08 | 0.05 | 0.05 | -0.40 | 0.08 | 0.00 |
|  | **Mortality** | **9** | -3 | 0.2 | 0.02 | -0.03 | -0.04 | -0.05 | 0.01 | 0.01 | -0.01 | -0.01 | **0.03** | 0.02 | -0.15 | -0.10 | 0.08 | 0.01 | 0.10 | -0.09 | 0.08 | -0.10 | -0.05 | 0.05 | -0.03 | 0.05 |
| **Group cage** | **Laying performance 1** | **10** | 2 | 18.9 | . | . | . | . | . | . | . | . | . | **0.24** | 0.15 | 0.10 | -0.25 | -0.06 | -0.10 | -0.15 | 0.60 | -0.07 | -0.05 | -0.10 | 0.05 | 0.05 |
|  | **Laying performance 2** | **11** | 3 | 6.2 | . | . | . | . | . | . | . | . | . | 0.10 | **0.10** | 0.70 | -0.20 | -0.05 | -0.10 | -0.05 | 0.09 | 0.40 | 0.08 | -0.10 | -0.04 | -0.10 |
|  | **Laying performance 3** | **12** | 3 | 7.5 | . | . | . | . | . | . | . | . | . | 0.15 | 0.80 | **0.10** | -0.20 | -0.03 | -0.15 | -0.05 | 0.08 | 0.50 | 0.08 | -0.08 | -0.05 | -0.08 |
|  | **Egg weight** | **13** | 1 | 2.9 | . | . | . | . | . | . | . | . | . | -0.30 | -0.05 | -0.08 | **0.70** | -0.20 | -0.05 | 0.10 | -0.04 | -0.30 | -0.15 | 0.85 | -0.15 | 0.01 |
|  | **Egg shell strength** | **14** | 2 | 6.2 | . | . | . | . | . | . | . | . | . | 0.05 | 0.03 | 0.05 | -0.05 | **0.35** | -0.04 | 0.11 | 0.05 | -0.05 | -0.05 | -0.15 | 0.78 | -0.03 |
|  | **Mortality** | **15** | -3 | 0.2 | . | . | . | . | . | . | . | . | . | -0.09 | -0.20 | -0.30 | -0.01 | 0.01 | **0.05** | 0.05 | 0.02 | -0.14 | 0.03 | 0.15 | 0.01 | 0.20 |
|  | **Feathering quality** | **16** | 3 | 1.5 | . | . | . | . | . | . | . | . | . | -0.10 | -0.08 | -0.09 | -0.02 | 0.01 | 0.09 | **0.40** | -0.05 | 0.04 | 0.04 | -0.02 | 0.02 | 0.08 |
| **Pract. environment** | **Laying performance 1** | **17** | 1 | 18.5 | . | . | . | . | . | . | . | . | . | . | . | . | . | . | . | . | **0.26** | 0.15 | -0.10 | -0.21 | 0.05 | -0.05 |
|  | **Laying performance 2** | **18** | 2 | 5.5 | . | . | . | . | . | . | . | . | . | . | . | . | . | . | . | . | 0.40 | **0.08** | 0.65 | -0.15 | 0.04 | -0.10 |
|  | **Laying performance 3+4** | **19** | 4 | 6.2 | . | . | . | . | . | . | . | . | . | . | . | . | . | . | . | . | 0.18 | 0.77 | **0.10** | -0.13 | 0.05 | -0.03 |
|  | **Egg weight** | **20** | 2 | 2.7 | . | . | . | . | . | . | . | . | . | . | . | . | . | . | . | . | -0.17 | -0.06 | -0.09 | **0.60** | -0.21 | 0.02 |
|  | **Egg shell strength** | **21** | 3 | 5.0 | . | . | . | . | . | . | . | . | . | . | . | . | . | . | . | . | -0.06 | -0.04 | -0.01 | -0.08 | **0.29** | -0.05 |
|  | **Mortality** | **22** | -5 | 0.2 | . | . | .. | . | . | . | . | . | . | . | . | . | . | . | . | . | -0.10 | -0.15 | -0.08 | 0.02 | -0.02 | **0.08** |

ew: relative economic weight per trait unit; SD; phenotypic standard deviation; heritabilities (diagonal), genetic correlations (above diagonal) and phenotypic correlations (below diagonal)
